# Generation and characterization of a mouse model of Becker muscular dystrophy with a deletion of *Dmd* exons 52 to 55

**DOI:** 10.1242/dmm.050595

**Published:** 2025-09-29

**Authors:** Lucie O. M. Perillat, Tatianna W. Y. Wong, Eleonora Maino, Abdalla Ahmed, Ori Scott, Elzbieta Hyatt, Paul Delgado-Olguin, Shagana Visuvanathan, Evgueni A. Ivakine, Ronald D. Cohn

**Affiliations:** ^1^ Department of Molecular Genetics, University of Toronto, Toronto, ON M5S 1A8, Canada; ^2^ Program in Genetics and Genome Biology, The Hospital for Sick Children Research Institute, Toronto, ON M5G 0A4, Canada; ^3^ Department of Translational Medicine, The Hospital for Sick Children, Toronto, ON M5G 1EB, Canada; ^4^ Institute of Medical Science, University of Toronto, Toronto, ON M5S 1A1, Canada; ^5^ Division of Immunology and Allergy, Department of Paediatrics, Hospital for Sick Children and University of Toronto, Toronto, ON M5S 1A1, Canada; ^6^ Heart & Stroke Richard Lewar Centre of Excellence, Toronto, ON M5S 3H2, Canada; ^7^ Department of Pediatrics, The Hospital for Sick Children, Toronto, ON M5G 1EB, Canada

**Keywords:** Becker muscular dystrophy, Duchenne muscular dystrophy, In-frame deletions, Mouse model, Muscular dystrophy, CRISPR-Cas9

## Abstract

Becker muscular dystrophy (BMD) is a rare X-linked recessive neuromuscular disorder, frequently caused by in-frame deletions in the *DMD* gene that result in the production of a truncated, yet functional, dystrophin protein. The consequences of BMD-causing in-frame deletions on the organism are difficult to predict, especially in regard to long-term prognosis. Here, we used CRISPR-Cas9 to generate a new *Dmd* Δ52-55 mouse model by deleting exons 52-55 in the *Dmd* gene, resulting in a BMD-like in-frame deletion. To delineate the long-term effects of this deletion, we studied these mice over 52 weeks by performing histology and echocardiography analyses and assessing motor functions. Our results suggest that truncated dystrophin is sufficient to maintain wildtype-like muscle and heart histology and functions in young mice. However, the truncated protein appeared to be insufficient to maintain normal muscle homeostasis and protect against exercise-induced damage at 52 weeks. To further delineate the effects of this exon 52-55 in-frame deletion, we performed RNA sequencing pre- and post-exercise and identified several differentially expressed pathways that reflect the abnormal muscle phenotype observed at 52 weeks in the BMD model.

## Teaser

**Summary:** Characterization of the long-term effects of a Becker muscular dystrophy (BMD)-like in-frame deletion of *Dmd* exons 52-55 in mice sheds new light on BMD pathology and disease mechanisms.

## INTRODUCTION

Becker muscular dystrophy (BMD) is a rare X-linked recessive neuromuscular disorder, frequently caused by in-frame deletions in the *DMD* gene that result in the production of a truncated (or internally deleted), but functional, dystrophin protein. Dystrophin is an essential component of the dystrophin glycoprotein complex (DGC) that is located in the sarcolemma and includes several components such as α-syntrophin (encoded by *Snta1* in mice), nNOS (encoded by *Nos1*) and β-sarcoglycan (encoded by *Sgcb*) (Duan et al., 2021). The DGC is required for muscle contraction and integrity (Fatehi et al., 2023). BMD is often considered as a milder form of Duchenne muscular dystrophy (DMD), in which variants in the *DMD* gene, including large deletions and duplications, result in the disruption of the reading frame and the malfunction or loss of the dystrophin protein (Bushby et al., 1991). Boys affected by DMD progressively lose their skeletal and cardiac muscular abilities, and require wheelchairs, ventilation and additional types of life support measures in their teenage years (Duan et al., 2021; Bello et al., 2016). Although BMD tends to be milder than DMD, patients with BMD present with a wide spectrum of clinical presentations, ranging from asymptomatic to an early loss of ambulation (Bushby et al., 1991; Nigro et al., 1994). Levels of dystrophin expression vary among patients with BMD and appear to correlate, to some extent, with disease severity (van den Bergen et al., 2014). This differential expression is also seen between muscles or even between different regions of the same muscle in individual patients (Heier et al., 2023). This variability remains largely misunderstood but is thought to arise from variable regenerative capacities, levels of inflammation, and the quantity and quality of dystrophin (van den Bergen et al., 2014). Worldwide, the prevalence of DMD and BMD is one in 3500-5000 boys (Walter and Reilich, 2017) and one in 18,500 boys (Bushby et al., 1991), respectively. Although significant therapeutic advances have been made in the context of DMD, BMD is, generally speaking, significantly less studied and understood (Heier et al., 2023).

A major barrier in understanding disease mechanism has been the lack of appropriate BMD animal models. The first mouse model of BMD, referred to as the *bmx* mouse model, was reported in 2023 by Heier and colleagues who used CRISPR-Cas9 technology to generate a 40,000-bp in-frame deletion of exons 45-47 in the *Dmd* gene (Heier et al., 2023). The *bmx* model appears to accurately recapitulate some of the phenotypes associated with BMD, including muscle weakness and heart dysfunction, and presents an intermediate phenotype between wildtype (WT) and *mdx52* model mice*,* recapitulating DMD (Heier et al., 2023). Most of the phenotypes reported in their paper were measured in young *bmx* mice (10 to 15 weeks old), with the exception of cardiac phenotypes, which were quantified in 18-month-old mice.

In the context of BMD, variants affecting different exons of the *DMD* gene have been shown to correlate with a range of phenotypes and disease severities (Echigoya et al., 2018). Consequently, there is substantial value in modeling and characterizing variants across various exons. This understanding led us to investigate another variant situated in the second mutational hotspot – specifically, the deletion of exons 52 to 55. Here, we used CRISPR-Cas9 to generate a new *Dmd* Δ52-55 mouse model that harbors a typical BMD-like in-frame deletion. We demonstrate that, at an early age, *Dmd* Δ52-55 mice are mostly indistinguishable from WT mice. At 12 weeks of age, *Dmd* Δ52-55 mice showed normal dystrophin expression and localization, normal muscle histology and mostly normal cardiac phenotypes. Functional tests also demonstrated that the truncated dystrophin protein expressed in *Dmd* Δ52-55 mice mostly maintains muscle function and protects against exercise-induced damage. However, some differences between *Dmd* Δ52-55 and WT mice seemed to appear as the mice grew older. Specifically, 52-week-old *Dmd* Δ52-55 mice showed reduced grip strength and contractile force after treadmill regimen. Therefore, our data suggest that a truncated dystrophin protein is insufficient to protect against exercise-induced damage at 52 weeks of age. To further delineate the effects of the *Dmd* exon 52-55 deletion, we performed an unbiased transcriptomic analysis pre- and post-exercise and identified several differentially expressed pathways, including the heat shock and ubiquitin responses as well as the BMP signaling pathway, that reflect the fatigued phenotype observed at 52 weeks in our model. Overall, the characterization of this newly generated mouse model of BMD sheds new light on BMD pathology and disease mechanisms.

## RESULTS

### Generation of the *Dmd* Δ52-55 mouse model with a BMD-like in-frame deletion using CRISPR-Cas9

**Fig. 1.**
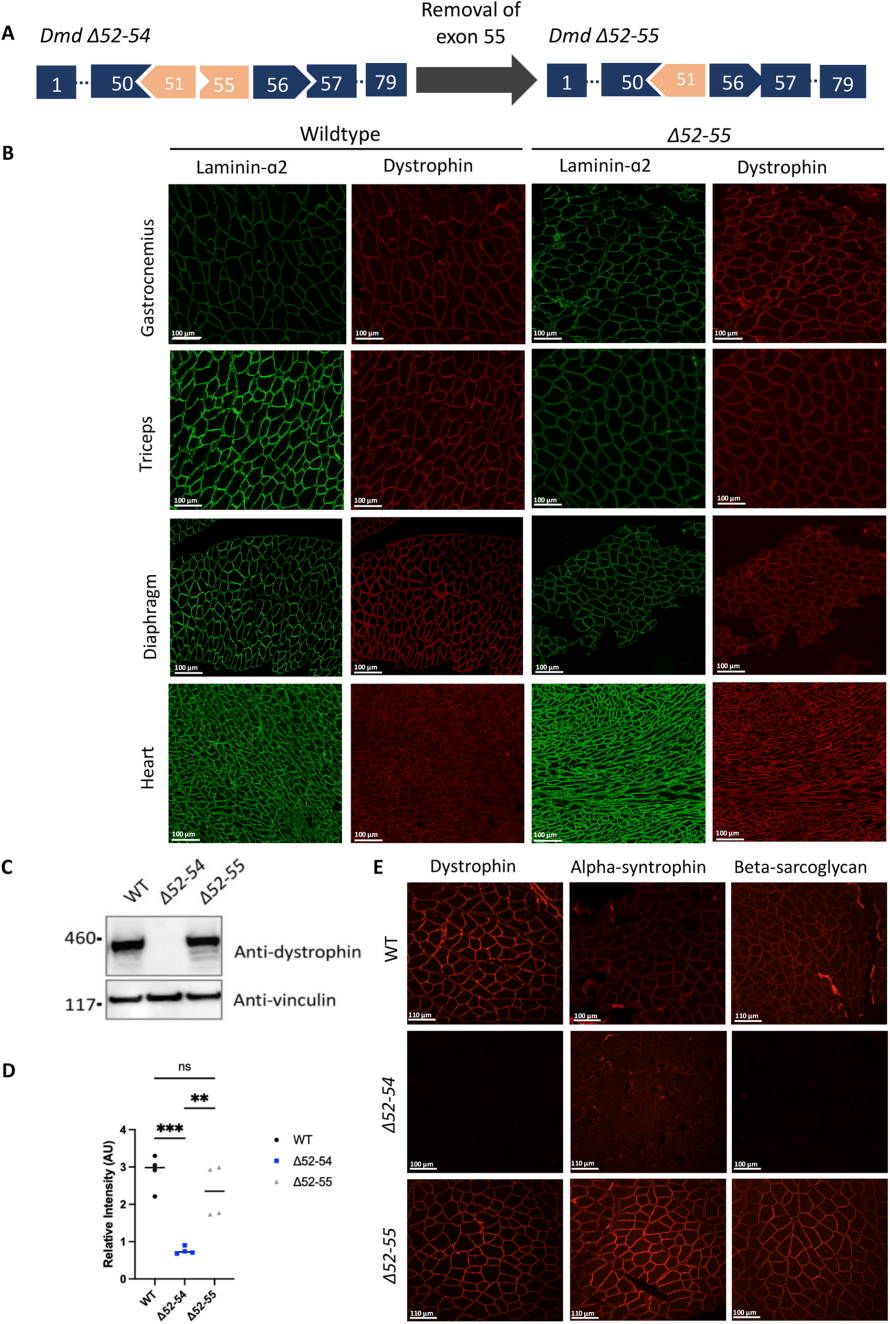
***Dmd* Δ52-55 mice have WT-like levels of truncated dystrophin expression, which maintains normal DGC localization to the sarcolemma.** (A) Schematic of the *Dmd* gene showing the Duchenne muscular dystrophy (DMD)-like deletion of exons 52 to 54 (*Dmd* Δ52-54) and the Becker muscular dystrophy (BMD)-like in-frame deletion of exons 52 to 55 (*Dmd* Δ52-55). Both deletions occur in the central rod domain of the dystrophin protein. (B) Immunofluorescence staining detected the sarcolemma using antibodies against laminin-α2 (encoded by *Lama2*, green) and dystrophin (red) in cross-sections of the gastrocnemius, triceps, diaphragm and heart from 12-week-old WT and *Dmd* Δ52-55 mice. Images represent data from five mice per genotype. Scale bars: 100 μm. (C) Western blotting of protein isolated from heart tissues showed dystrophin expression in the *Dmd* Δ52-55 mouse model but not in the DMD mouse model *Dmd* Δ52-54. Vinculin was used as a loading control. (D) Quantification of relative intensity [dystrophin/DAPI, arbitrary units (AU)] from immunofluorescence staining in gastrocnemius of 12-week-old WT (*n*=4), *Dmd* Δ52-54 (*n*=4) and *Dmd* Δ52-55 (*n*=4) mice. Bars show the mean. Statistical analyses were performed with one-way ANOVA with Tukey's post hoc test. ns, not significant; **P<0.01; ***P<0.001. (E) Immunofluorescence staining against dystrophin, α-syntrophin and β-sarcoglycan (components of the dystrophin glycoprotein complex or DGC) in the gastrocnemius of 12-week-old WT, *Dmd* Δ52-54 mice and *Dmd* Δ52-55 mice. Images represent data from five mice per genotype. Scale bars: 100 or 110 μm as indicated. The WT and *Dmd* Δ52-54 mouse data shown in panels B,C,E have been previously reported in figure 2 of Wong et al. (2020). The uncropped blots showing *Dmd* Δ52-55 protein levels in addition to WT and *Dmd* Δ52-54 protein levels in C are reproduced from figure 2B of Wong et al. (2020) and the α-syntrophin panel for *Dmd* Δ52-54 in E is reproduced from figure 2D of Wong et al. (2020) under the terms of a CC-BY 4.0 licence.

The deletion of exons 52 to 55 is a representative candidate variant for generating a BMD-like in-frame deletion as it lies within one of the known mutational hotspots in the *DMD* gene (Fig. 1A). To generate our new mouse model of a BMD-causing in-frame deletion, we designed four single guide RNAs (sgRNAs) that remove a 213 kb region spanning *Dmd* exons 52 to 55. Two sgRNAs targeting both introns 51 and 55 were designed to increase the frequency of editing events (Fig. S1A). The sgRNAs and *Streptococcus pyogenes* Cas9 mRNA were delivered into fertilized zygotes through pronuclear injections and implanted in pseudopregnant females. The F0 mouse pups were genotyped for the presence of the predicted deletion junction, which was identified in one pup out of 25. Sanger sequencing also identified a deletion of an additional 5 bp upstream and downstream of the expected Cas9 cut sites (Fig. S1B,C). The absence of exons 52 to 55 was confirmed through whole-genome sequencing (Fig. S1D). The splice sites were unaffected as validated by reverse transcription PCR (Fig. S1E). These findings thus confirm the generation of a *Dmd* Δ52-55 mouse model with a *Dmd* in-frame deletion.

### *Dmd* Δ52-55 mice have WT-like dystrophin expression and localization at 12 weeks of age

Patients with DMD and BMD typically present with a lack or reduction of expression of dystrophin in muscle tissues (Duan et al., 2021; Bello et al., 2016; Heier et al., 2023). To understand the implications of a BMD-like in-frame deletion on the expression of dystrophin, we imaged and quantified dystrophin expression levels in 12-week-old mice. Of important note, our *Dmd* Δ52-55 mice were compared to *Dmd* Δ52-54 mice that harbor a complete DMD phenotype (characterized in Wong et al., 2020) as well as WT mice. The WT, *Dmd* Δ52-54 and *Dmd* Δ52-55 mouse data reported here and in Wong et al. (2020) were obtained concurrently within the same sets of experiments, and some datasets for WT and *Dmd* Δ52-54 mice from Wong et al. (2020) are reproduced here as indicated in the figure legends. As shown in Fig. 1B-E, dystrophin expression levels were similar in *Dmd* Δ52-55 and WT mice in different muscle groups at 12 weeks of age. Immunofluorescence staining indicated that dystrophin expressed in *Dmd* Δ52-55 mice localized correctly to the sarcolemma in the gastrocnemius alongside α-syntrophin and β-sarcoglycan, two components of the DGC (Fig. 1B,E). These findings indicate that 12-week-old *Dmd* Δ52-55 mice have WT-like levels of truncated dystrophin expression, which maintains normal DGC localization to the sarcolemma at 12 weeks.

### *Dmd* Δ52-55 mice present WT-like muscle histology at 12 weeks of age

**Fig. 2.**
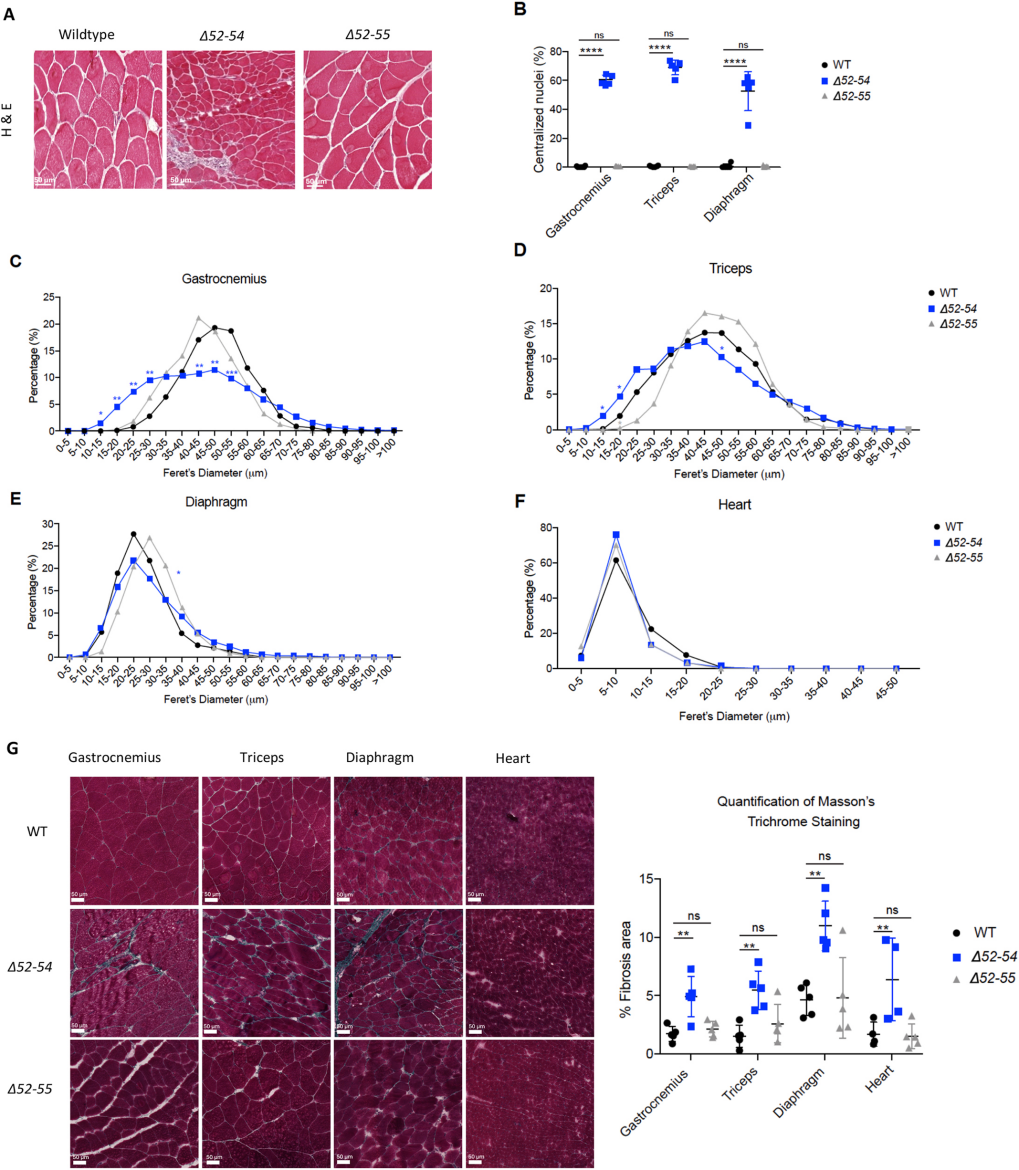
***Dmd* Δ52-55 mice present WT-like muscle histology.** (A) Hematoxylin and Eosin (H&E) staining of gastrocnemius cross-sections from 12-week-old WT, *Dmd* Δ52-54 and *Dmd* Δ52-55 mice. Scale bars: 50 µm. (B) Quantification of centralized nuclei in 12-week-old WT (*n*=6), *Dmd* Δ52-54 (*n*=5) and *Dmd* Δ52-55 (*n*=5) mice. (C-F) Distribution of minimum Feret's diameter quantified using H&E cross-sections from WT (*n*=5), *Dmd* Δ52-54 (*n*=5) and *Dmd* Δ52-55 (*n*=5) mice in the gastrocnemius (C), triceps (D), diaphragm (E) and heart (F). (G) Masson's trichome staining was performed on gastrocnemius, triceps, diaphragm and heart tissues of 12-week-old WT (*n*=5), *Dmd* Δ52-54 (*n*=4-5) and *Dmd* Δ52-55 (*n*=5) mice and the fibrotic area was quantified. Scale bars: 50 μm. All data are represented as the mean±s.d. Statistical analyses were performed with two-tailed unpaired Student's *t*-test. ns, not significant; *P<0.05; **P<0.01; ***P<0.001; ****P<0.0001. The WT and *Dmd* Δ52-54 mouse data shown in panels B-E and G have been previously reported in figure 3 of Wong et al. (2020) and the quantification data are reproduced here alongside *Dmd* Δ52-55 mouse data.

Patients with DMD and BMD experience progressive muscle deterioration, which is characterized in Hematoxylin and Eosin (H&E)-stained tissue sections by: (1) immature myofibers containing centralized nuclei, (2) heterogeneity in muscle fiber sizes and (3) development of fibrotic tissue (Briguet et al., 2004). To determine the effects of the in-frame deletion of *Dmd* exons 52-55 on muscle degeneration and regeneration, we compared the results for our *Dmd* Δ52-55 mice to data for WT and *Dmd* Δ52-54 mice (which have been previously reported in Wong et al., 2020). We first performed H&E staining on the gastrocnemius of 12-week-old WT, *Dmd* Δ52-54 and *Dmd* Δ52-55 mice (Fig. 2A) and quantified centralized nuclei in the gastrocnemius, triceps and diaphragm of 12-week-old mice (Fig. 2B). We observed less than 1% of myofibers with centralized nuclei in *Dmd* Δ52-55 mice, which is a level comparable to that observed in WT mice (Fig. 2B). These results indicate that mice with a BMD-like in-frame deletion of *Dmd* exons 52 to 55 show normal levels of muscle turnover at 12 weeks of age.

The size of cross-sections of myofibers was then quantified based on minimal Feret's diameter in the gastrocnemius, triceps, diaphragm and heart of 12-week-old mice. Myofibers were found to be comparably homogeneous in size in WT and *Dmd* Δ52-55 mice across tissues, suggesting that muscle degeneration and regeneration occurs normally (Fig. 2C-F).

Next, the levels of fibrotic tissue were quantified using Masson's trichrome staining on the gastrocnemius, triceps, diaphragm and heart of 12-weeks-old mice. Fibrotic changes were not observed in *Dmd* Δ52-55 mice (Fig. 2G).

Taken together, these results indicate that a BMD-like in-frame deletion of *Dmd* exons 52 to 55 does not affect muscle histology and the levels of muscle degeneration and regeneration. At 12 weeks of age, we, therefore, see WT-like muscle histology in mice expressing a truncated dystrophin protein.

### *Dmd* Δ52-55 mice have WT-like cardiac phenotypes

**Fig. 3.**
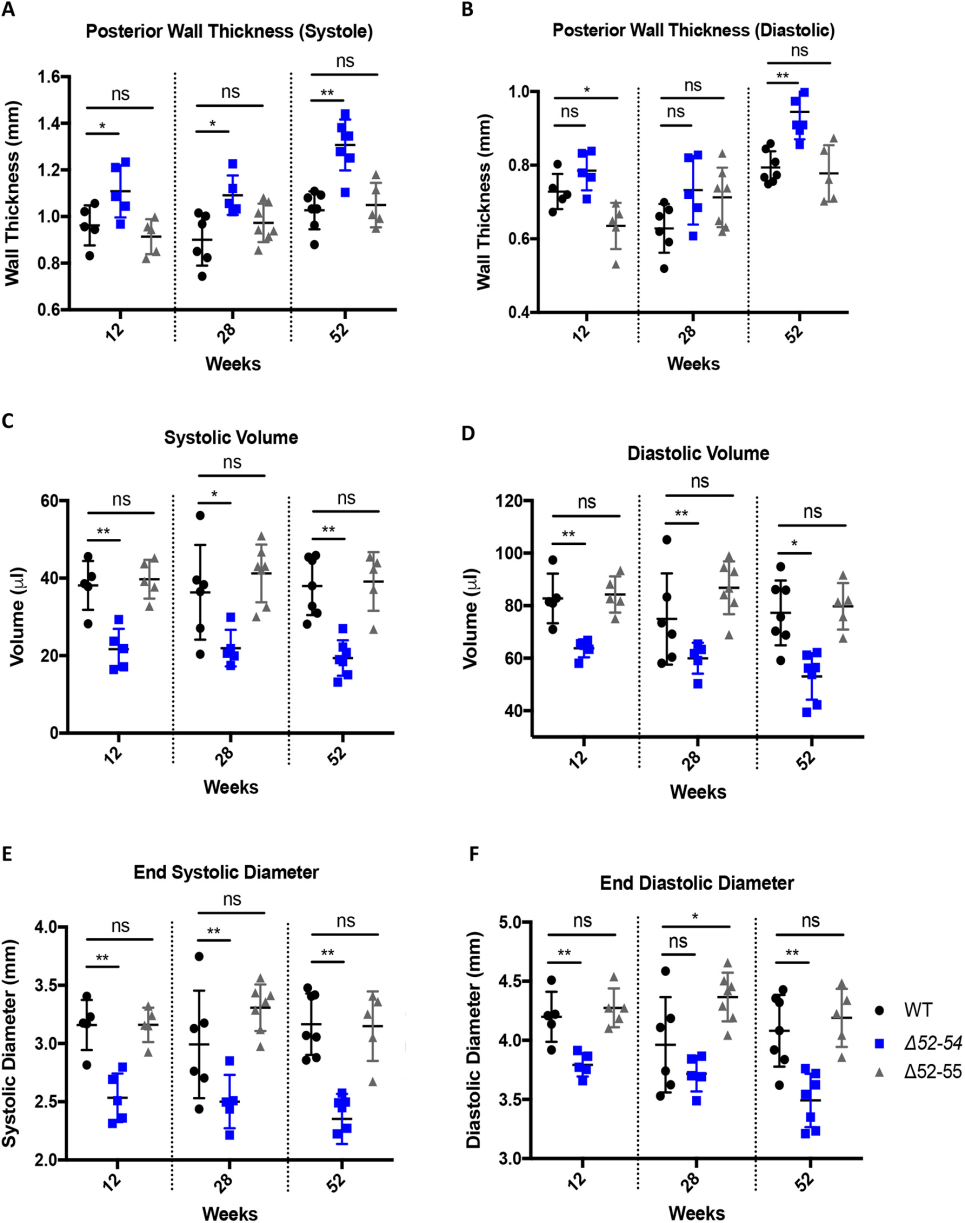
***Dmd* Δ52-55 mice maintain mostly WT-like cardiac anatomical features throughout 12 to 52 weeks of age.** Hearts in WT (*n*=5-7), *Dmd* Δ52-54 (*n*=5-7) and *Dmd* Δ52-55 (*n*=5-7) mice were analyzed using echocardiography to determine left ventricular posterior wall thickness during systole (A) and diastole (B), left ventricular systolic (C) and diastolic (D) volume, and left ventricular end systolic (E) and diastolic (F) diameter. All data are represented as mean±s.d. Statistical analyses were performed with two-tailed unpaired Student's *t*-test. ns, not significant; *P<0.05; **P<0.01. The WT and *Dmd* Δ52-54 mouse data shown in panels A-F have been previously reported in figures 6 and S1 of Wong et al. (2020) and are reproduced here alongside *Dmd* Δ52-55 mouse data.

Patients with DMD and BMD tend to present with progressive cardiac hypertrophy, arrhythmia or dilation of the ventricles due to the reduction of dystrophin expression in cardiac tissues (Hermans et al., 2010; Heier et al., 2023). Echocardiography results showed that our *Dmd* Δ52-55 model presented with similar posterior wall thickness during systole, systolic volume, diastolic volume and end systolic diameter as those of WT mice throughout 12 to 52 weeks of age (Fig. 3A,C-E). We saw a small, yet significant increase in posterior wall thickness during diastole in *Dmd* Δ52-55 mice compared to that in WT mice at 12 weeks of age (Fig. 3B) and a decreased end diastolic diameter at 28 weeks of age (Fig. 3F). In Fig. 3A-F, results from our *Dmd* Δ52-55 mice were compared to previously published results for *Dmd* Δ52-54 and WT mice (Wong et al., 2020).

*Nppa* expression levels were measured in *Dmd* Δ52-55 mice as a proxy for cardiac stress (Giovou et al., 2024). *Dmd* Δ52-55 mice had similar levels of *Nppa* expression to those in WT mice (Fig. S2A). The heart rate in *Dmd* Δ52-55 mice was also similar to that in WT throughout 12 to 52 weeks of age, indicating an absence of tachycardia in mice expressing the truncated dystrophin protein (Fig. S2B). The ejection fraction and fractional shortening were similar between *Dmd* Δ52-55 and WT mice (Fig. S2C,D). Here again, results from our *Dmd* Δ52-55 mice were compared to previously published results for *Dmd* Δ52-54 and WT mice (Wong et al., 2020). Overall, *Dmd* Δ52-55 mice expressing a truncated dystrophin protein maintained mostly normal cardiac parameters similar to those in WT mice, indicating limited cardiac stress in these mice.

### Truncated dystrophin expressed in *Dmd* Δ52-55 mice maintains muscle function and protects against exercise-induced damage at 12 weeks of age

**Fig. 4.**
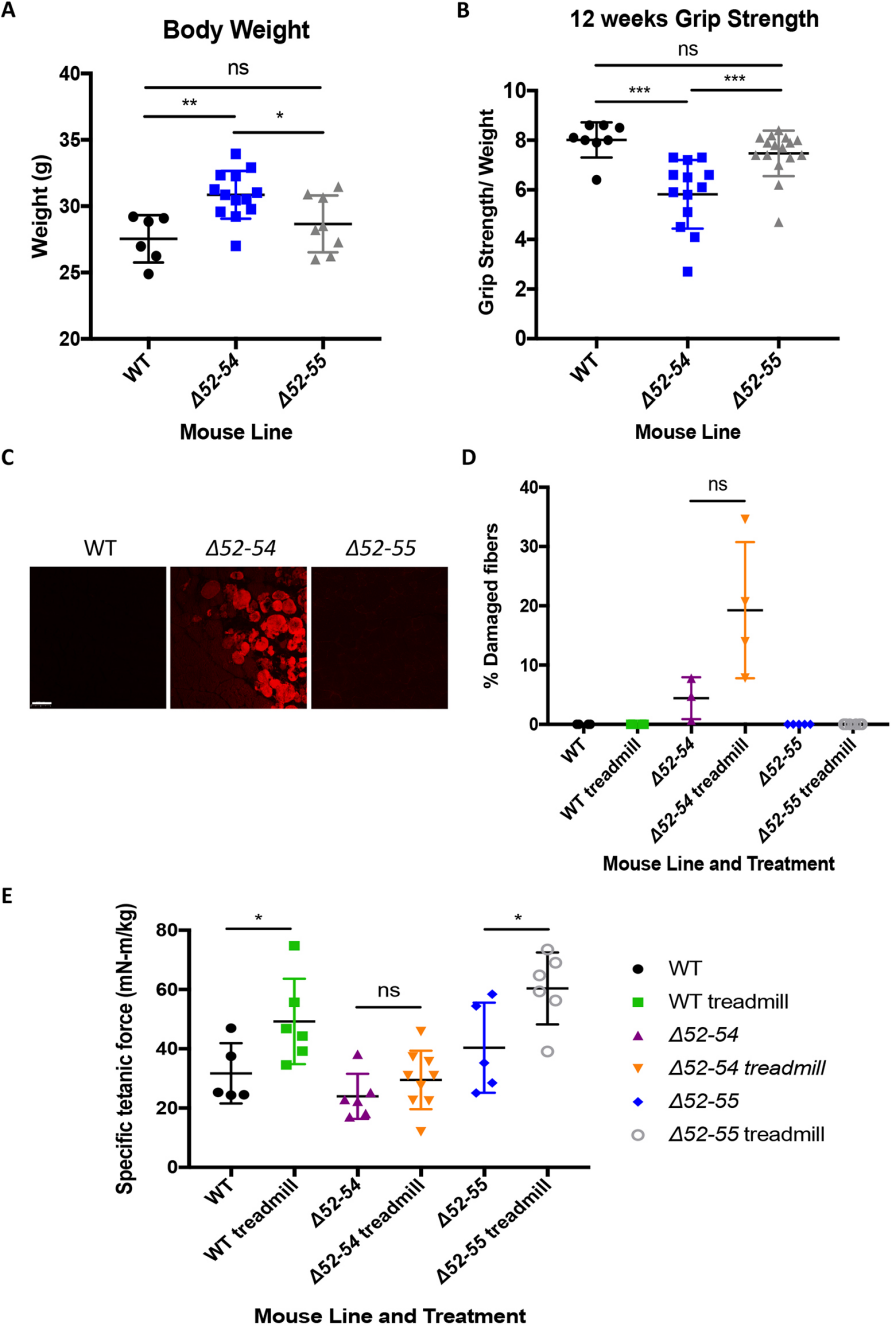
**Truncated dystrophin expressed in *Dmd* Δ52-55 mice is not detrimental to muscle function and protects from muscle aggravation at 12 weeks.** (A) Body weight of WT (*n*=6), *Dmd* Δ52-54 (*n*=13) and *Dmd* Δ52-55 (*n*=8) mice at 12 weeks of age. (B) Forelimb and hindlimb grip strength of 12-week-old WT (*n*=8), *Dmd* Δ52-54 (*n*=13) and *Dmd* Δ52-55 (*n*=16) mice. (C) Gastrocnemius cross-sections from exercised WT, *Dmd* Δ52-54 and *Dmd* Δ52-55 12-week-old mice injected with Evan's Blue dye (EBD). Scale bar: 100 μm. (D) EBD-positive myofibers in gastrocnemius of sedentary and exercised WT, *Dmd* Δ52-54 and *Dmd* Δ52-55 mice were quantified. (E) Tetanic force was measured in 12-week-old sedentary and exercised WT, *Dmd* Δ52-54 and *Dmd* Δ52-55 mice. All data are represented as mean±s.d. Statistical analyses were performed with two-tailed unpaired Student's *t*-test. ns, not significant; *P<0.05; **P<0.01; ***P<0.001. The WT and *Dmd* Δ52-54 mouse data shown in panels A,B have been previously reported in figure 5 of Wong et al. (2020) and are reproduced here alongside *Dmd* Δ52-55 mouse data.

In DMD and BMD, the presence of fibrosis typically impairs muscle function and regeneration capacity (Capitanio et al., 2020). To assess the ability of the truncated dystrophin protein expressed in *Dmd* Δ52-55 mice to protect against exercise-induced muscle damage, several functional tests were performed, including grip strength and tetanic force after treadmill regimen. At 12 weeks of age, *Dmd* Δ52-55 mice showed WT-like weight (Fig. 4A) and grip strength (Fig. 4B). *Dmd* Δ52-55 mice showed no areas with damaged fibers before or after the treadmill regimen, unlike *Dmd* Δ52-54 mice, which showed higher numbers of damaged fibers as evidenced by Evan's Blue dye (EBD) uptake (Fig. 4C,D). Tetanic force was also measured in sedentary and exercised *Dmd* Δ52-55 mice and indicated a similar response to exercise between *Dmd* Δ52-55 and WT mice, namely, an increase in muscle strength after exercise (Fig. 4E). This is markedly different from the response seen in *Dmd* Δ52-54 mice, which showed no increase in tetanic force after exercise. Although *Dmd* Δ52-55 mice express a shorter dystrophin protein, there was no detriment to grip strength function at 12 weeks of age. Similarly to full-length dystrophin, the shorter dystrophin protein protected from muscle stimulation while allowing enhanced muscle strength after exercise.

### Truncated dystrophin expressed in *Dmd* Δ52-55 mice is not sufficient to maintain normal muscle function at 52 weeks of age

Although the truncated dystrophin protein expressed in *Dmd* Δ52-55 appeared to mostly maintain muscle function at 12 weeks of age, a few differences between *Dmd* Δ52-55 and WT mice [including smaller fibers observed in the gastrocnemius in *Dmd* Δ52-55 mice and some cardiac phenotypes such as posterior wall thickness (diastolic) and diastolic diamater (Fig. 3B,F)] prompted us to investigate muscle function in older, 52-week-old mice.

**Fig. 5.**
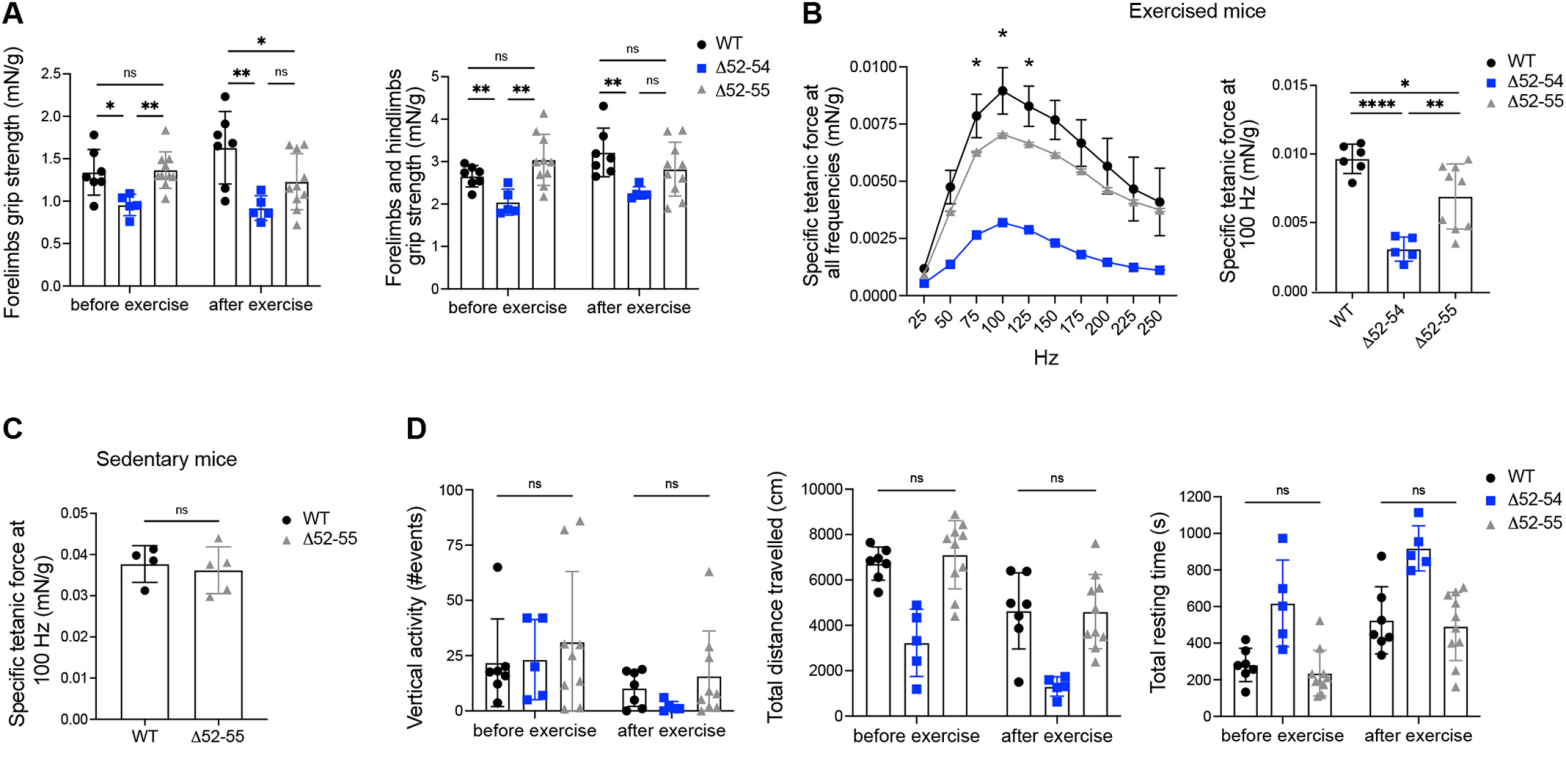
***Dmd* Δ52-55 mice show impaired muscle function at 52 weeks of age.** (A) Forelimb and combined forelimb and hindlimb grip strength in WT (*n*=7), *Dmd* Δ52-54 (*n*=5) and *Dmd* Δ52-55 (*n*=8) mice. (B) Contractile force in WT (*n*=6), *Dmd* Δ52-54 (*n*=5) and *Dmd* Δ52-55 (*n*=9) mice measured after treadmill exercise. (C) Contractile force in sedentary WT and *Dmd* Δ52-55 mice. (D) Open-field performances in vertical activity, total distance traveled and total resting time in WT (*n*=7), *Dmd* Δ52-54 (*n*=5) and *Dmd* Δ52-55 (*n*=9-10) mice. All data are represented as mean±s.d. Statistical analyses were performed with two-tailed unpaired Student's *t*-test. ns, not significant; *P<0.05; **P<0.01; ****P<0.0001.

At 52 weeks of age, forelimb grip strength was lower in *Dmd* Δ52-55 mice compared to that in WT mice after treadmill exercise (Fig. 5A). *Dmd* Δ52-55 mice also showed a decrease in contractile force at 75, 100 and 125 Hz after exercise compared to WT mice (Fig. 5B,C). Although differences were observed in grip strength and contractile force at 52 weeks of age, performances in open-field parameters were similar between *Dmd* Δ52-55 and WT mice pre- and post-exercise (Fig. 5D). These data suggest that a truncated dystrophin protein is not sufficient to maintain fully WT-like muscle function with age.

### Exercise induces differential gene expression in *Dmd* Δ52-55 mice at 52 weeks of age

**Fig. 6.**
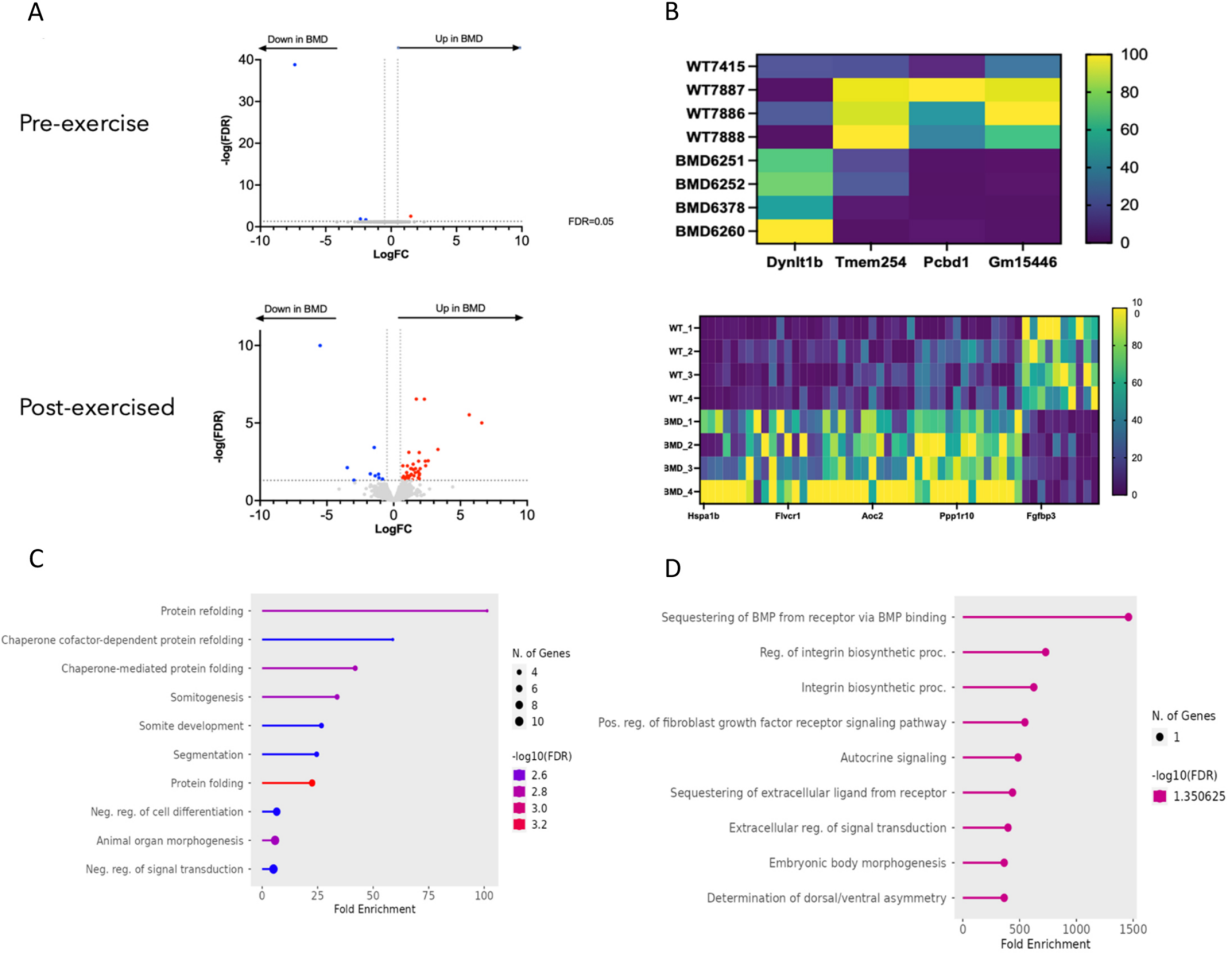
**Exercise induces differential gene expression in the gastrocnemius of 52-week-exercised *Dmd* Δ52-55 mice.** (A) Volcano plots showing genes that are upregulated (in red) and downregulated (in blue) in *Dmd* Δ52-55 mice (*n*=4) compared to WT mice (*n*=4) before and after exercise. FC, fold change; FDR, false discovery rate. (B) Heat maps showing genes that are upregulated and downregulated in *Dmd* Δ52-55 mice (*n*=4) compared to WT mice (*n*=4) before (top) and after (bottom) exercise. (C,D) Gene Ontology analysis showing the distribution of Gene Ontology terms significantly upregulated (C) and downregulated (D) between *Dmd* Δ52-55 and WT mice. neg., negative; pos., positive; proc., process; reg., regulation.

Given the lower grip strength and contractile force observed in 52-week-old-exercised *Dmd* Δ52-55 mice, we hypothesized that exercise aggravated the fatigue phenotype by inducing transcriptional changes involved in muscle regeneration and degeneration. We performed RNA sequencing on the gastrocnemius of 52-week-old sedentary and exercised BMD-like mice (*Dmd* Δ52-55), DMD mice (*Dmd* Δ52-54) and WT mice. *Dmd* Δ52-54 mice were used as a reference and behaved as expected, with a large number of differentially expressed genes compared to WT mice both pre- and post- exercise (Fig. S3). Genes encoding subunits and interactors of the dystrophin-associated protein complex (DAPC) were differentially expressed in 52-week-old *Dmd* Δ52-54 mice; specifically, *Sntb1*, *Dtna1*, *Nos3*, *Actb* amd *Lama4* were upregulated, whereas *Nos1* and *Lama5* were downregulated in both exercised and non-exercised 52-week-old *Dmd* Δ52-54 mice. In contrast, no subunits of the DAPC were found to be upregulated or downregulated in our *Dmd* Δ52-55 model at 52 weeks of age. Although only four genes were differentially expressed between the BMD-like (*Dmd* Δ52-55) and WT cohorts of sedentary mice, 53 genes were upregulated or downregulated in exercised *Dmd* Δ52-55 mice compared to WT mice. Some of these genes, including *Hspa1a* and *Hspa1b*, encoding protein-folding chaperones, were substantially upregulated (97- and 50-fold change in expression, respectively). These data suggest that exercise induces significant transcriptional changes in *Dmd* Δ52-55 mice (Fig. 6A,B).

To better understand which pathways are involved, we performed Gene Ontology and gene set enrichment analyses for each cohort. As expected for *Dmd* Δ52-54 mice, upregulated gene pathways included those involved in inflammation, wound healing and calcium signaling (dysfunction of calcium-dependent proteases), whereas downregulated pathways included those involved in cytoskeletal organization (especially in exercised mice), the release of sequestered Ca^2+^ ions and calcium binding (acting upstream of the actin cytoskeleton reorganization), and muscle and heart contraction and development (Fig. S3). We then identified differentially expressed genes in the *Dmd* Δ52-55 exercised mice compared with WT exercised mice (Fig. 6C,D). Upregulated pathways included protein folding, clearance of protein aggregation, heat shock and ubiquitin responses and the organization of the cytoskeleton, whereas downregulated genes were involved in BMP signaling or belonged to the Gm family of long non-coding RNAs [Fig. 6D; RNA-sequencing data uploaded to Zenodo (https://zenodo.org/records/17087788; https://zenodo.org/records/17107657; https://zenodo.org/records/17095147)]. Taken together, these results suggest that gene expression is influenced by exercise in *Dmd* Δ52-55 mice leading to the activation of catabolic pathways in skeletal muscles.

## DISCUSSION

Here, we generate and characterize a new mouse model of BMD, in which CRISPR-Cas9 technology was used to generate a BMD-like in-frame deletion of *Dmd* exons 52 to 55. This deletion is located in one of the two mutational hotspots in the *DMD* gene and is, therefore, likely to be relevant to patients with BMD. To our knowledge, this is the first mouse model of BMD in which phenotypes other than those affecting the heart were studied over a period of 52 weeks. We demonstrate that, at an early age, *Dmd* Δ52-55 mice are mostly identical to WT mice: at 12 weeks of age, *Dmd* Δ52-55 mice show normal dystrophin expression and localization, normal muscle histology and mostly normal cardiac phenotypes. Functional tests also demonstrate that the truncated dystrophin protein expressed in *Dmd* Δ52-55 mice mostly maintains muscle function and protects against exercise-induced damage. This is consistent with the fact that exons 52 to 55 are within the central rod domain of the dystrophin protein, thus leaving the essential actin-binding domain, cysteine-rich domain and C-terminal domain unaffected (Sun et al., 2020).

However, some differences between *Dmd* Δ52-55 and WT mice seem to appear as mice age and are aggravated by exercise. Exercised 52-week-old *Dmd* Δ52-55 mice showed reduced forelimb grip strength and contractile force compared to WT mice. Our unbiased transcriptomic data suggest that this fatigued phenotype is associated with a change in gene expression that appears to be induced by exercise. Looking more closely at the genes that are differentially expressed in *Dmd* Δ52-55 exercised mice may provide some preliminary insights into the mechanisms underlying this fatigued phenotype. Gene Ontology and gene set enrichment analyses revealed that genes involved in heat shock and ubiquitin responses are upregulated in our newly generated BMD mouse model following exercise. This trend has also been observed in DMD mice (Nishimura and Sharp, 2005; Bornman et al., 1995; Wattin et al., 2018). De Paepe and colleagues (2012) also note that heat shock proteins and chaperones balance muscle regeneration and destruction in patients with DMD. They analyzed muscle biopsies from seven patients with DMD and concluded that heat shock proteins are upregulated in regenerating atrophic muscles as well as in macrophages and cytotoxic T cells invading non-necrotic muscle fibers (De Paepe et al., 2012). A similar mechanism could exist in BMD, where the upregulation of heat shock proteins and chaperones would be present in both regenerating fibers and immune cells. Further work is required to determine whether this hypothesis is valid or whether the heat shock and ubiquitin responses are only observed in a single cell type, thereby promoting either protection or destruction. Interestingly, although extracellular matrix proteins were upregulated in the *bmx* model (Heier et al., 2023), no upregulation in collagen or fibrosis-related genes was observed in the *Dmd* Δ52-55 model at 52 weeks, whether pre- or post-exercise.

Gene Ontology and gene set enrichment analyses also identified genes that are downregulated in our newly generated BMD mouse model, namely, genes involved in BMP signaling and pertaining to the Gm family of long non-coding RNAs. In the context of BMP signaling, our RNA-sequencing data reveal that the expression of *Grem2*, encoding a BMP antagonist, is downregulated in our BMD-like *Dmd* Δ52-55 mice. Homeostatic BMP signaling is integral to muscle differentiation and regeneration capacity. Downregulation of *Grem2* has been observed in patients with DMD (Pescatori et al., 2007); the authors of this study analyzed genome-wide gene expression profiles of 19 patients with DMD and identified *Grem2* as one of the top 18 differentially expressed genes in muscles. Shi and colleagues also observed inefficient differentiation of myoblast in DMD mice models (Shi et al., 2011, 2013). Here, it could be hypothesized that the downregulation of *Grem2* leads to the upregulation of BMP signaling, which, in turns, prevents muscle cell differentiation and muscle regeneration in our BMD-like *Dmd* Δ52-55 mice. Further work is required to validate this hypothesis and determine whether this mechanism significantly contributes to BMD pathology. Furthermore, although it is known that some of the genes that belong to the Gm family of long non-coding RNAs mentioned earlier are involved in RNA polymerase II DNA-binding activity, little is known about this gene family overall. As such, further work would be necessary to shed light on their contribution to BMD pathology.

**Fig. 7.**
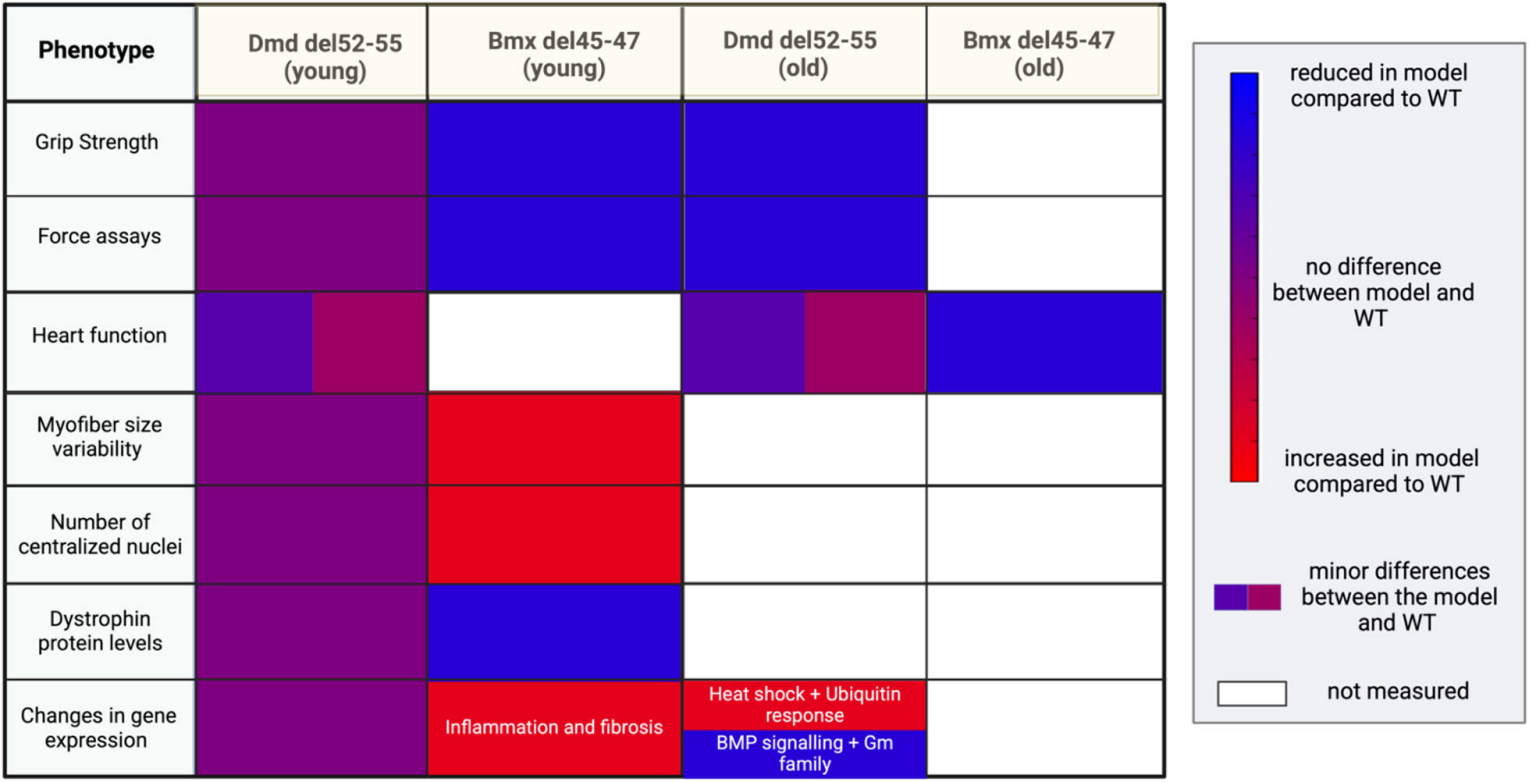
Comparisons of phenotypes observed in the *bmx* model and our *Dmd* Δ52-55 model.

As noted by Heier and colleagues (2023), it is important to generate more models of BMD in view of comparing them and identifying which dystrophin isoforms (and internal deletions) are associated with what phenotype. The latter is important for at least three reasons: (1) to provide better predictions about disease severity and phenotype to patients and their families, (2) to identify the effects of treatments for different BMD variants, and (3) to determine which patients with DMD have *DMD* variants that are more amenable to exon skipping and to predict the extent to which the dystrophic phenotype would be altered using this approach (Bello et al., 2016). Although deletions in the N- or C-terminal domains of the dystrophin protein typically result in a DMD phenotype, deletions in the rod domain of dystrophin may have variable effects based on the structural ‘phase’ between spectrin repeats (Bello et al., 2016; Duchêne et al., 2018). It has been observed that in-frame variants in exons 50-51 typically result in a milder BMD phenotype, whereas variants in exons 45-53 (excluding exons 50-51) usually lead to a more severe BMD phenotype (Bello et al., 2016). Little is known about variants in the remaining part of the mutational hotspot, namely exons 53-55. Our newly generated model of BMD addresses this knowledge gap. Results obtained with our *Dmd* Δ52-55 mouse model can be compared with those observed in the *bmx* mouse model (deletion of exons 45-47) generated by Heier and colleagues (2023), as shown in Fig. 7. Overall, it appears that the BMD phenotype is more severe in the *bmx* model than in our *Dmd* Δ52-55 model. This is consistent with our knowledge of dystrophin: the deletion of exons 45-47 is predicted to disrupt spectrin repeats and nNOS binding (Heier et al., 2023), thereby significantly disrupting the DGC. In the future, we hope that such comparisons can be made between different models of BMD in order to gain insights into the functionality of different dystrophin isoforms.

Treatment options for patients with BMD are very limited, even more so than for patients with DMD. This issue is exacerbated by the fact that very few clinical trials are currently being conducted for patients with BMD. Glucocorticoid treatment, which is the most widely used and accessible treatment option for patients with DMD, is rarely used in patients with BMD as the risk-benefit profile of this treatment for a longer BMD disease course remains unclear (Heier et al., 2023). Glucocorticoids have anti-inflammatory and immunosuppressive properties and were shown to delay loss of ambulation in patients with muscular dystrophies (Bello et al., 2016). However, glucocorticoids come with significant side effects, such as weight gain, adrenal suppression and mood imbalances (Heier et al., 2023). Recently, vamorolone, a new dissociative anti-inflammatory with an improved safety profile, has been approved by the US Food and Drug Administration (Heier et al., 2019). We hope that BMD mouse models and natural history studies will continue to be used in conjunction to further advance treatments and identify which therapeutic option is best according to the underlying variant exhibited in individual patients.

## MATERIALS AND METHODS

### Animal husbandry

All mice were housed at The Centre for Phenogenomics, Toronto, Ontario, Canada, under the environmental regulations of a 12 h/12 h light/dark cycle with food and water provided in individual units (Techniplast). All procedures were conducted in accordance with the Animals for Research Act of Ontario and the Guidelines of the Canadian Council on Animal Care. Animal protocols were performed at The Center for Phenogenomics and were reviewed and approved by the local Animal Care Committee. All experiments were performed by operators who were unaware of genotype and experimental conditions. Mice were randomly assigned to the ‘sedentary’ or ‘exercised’ experimental group. No mice were excluded from the analysis. WT, *Dmd* Δ52-54 and *Dmd* Δ52-55 mouse data were obtained concurrently within the same sets of experiments, and some results from these datasets for WT and *Dmd* Δ52-54 mice have been previously published in Wong et al. (2020), which are indicated under each figure, as appropriate.

### sgRNA design

sgRNAs specific for the *S. pyogenes* Cas9 system were designed using Benchling (https://www.benchling.com/) (Table S1). All sgRNAs were synthesized following Gertsenstein and Nutter (2018).

### Pronuclear injections

Three- to 4-week-old C57BL/6J (The Jackson Laboratory) female mice were used as embryo donors. Pseudopregnant surrogates were CD-1 (ICR) (Charles River) females. All procedures were conducted following Behringer et al. (2014). Briefly, embryo donors were superovulated and mated overnight with males. Successfully mated females were selected and the oviducts were dissected to isolate fertilized zygotes. Zygotes were then subjected to pronuclear microinjections with sgRNAs and Cas9 mRNA. Each injection comprised 10 ng/μl of each sgRNA and 20 ng/μl of Cas9 mRNA. Injected embryos were transferred into the oviducts of the surrogate females. Mice were backcrossed for three generations and then intercrossed before used for analysis. Only males were used in this study. Pronuclear injections for *Dmd* Δ52-54 and *Dmd* Δ52-55 mice were performed concurrently within the same sets of experiments. Characterization of *Dmd* Δ52-54 mice has been previously published in Wong et al. (2020), as indicated in the figure legends.

### DNA extraction and analyses

Mouse tails were biopsied at 2 weeks of age, and DNA was extracted using a DNAeasy Blood and Tissue kit (Qiagen). 1 μl of the extracted DNA was used for PCR with DreamTaq polymerase (Thermo Fisher Scientific) and the primers are shown in Table S2. The DNA extraction and analyses for *Dmd* Δ52-54 and *Dmd* Δ52-55 mice were performed concurrently within the same sets of experiments, and some results from these datasets for WT and *Dmd* Δ52-54 mice have been previously published in Wong et al. (2020).

### Sanger sequencing

Sanger sequencing was performed using an Applied Biosystems SeqStudio Genetic Analyser, the BigDye Terminator version 3.1 Cycle Sequencing Kit (Thermo Fisher Scientific) and the BigDye xTerminator Purification Kit (Thermo Fisher Scientific), following the manufacturers' protocols. Electropherograms were analyzed using SnapGene (https://www.snapgene.com).

### RT-PCR

TRIzol reagent (Thermo Fisher Scientific) was used for RNA extraction according to the manufacturer's instructions. Reverse transcription of 1 μg of mRNA was performed using the SuperScript III reverse transcriptase kit (Thermo Fisher Scientific).

### Whole-genome sequencing

DNA extracted from mouse tails was used for whole-genome sequencing, which was performed using the Illumina HiSeq X system (San Diego, CA, USA) by The Centre for Applied Genomics at the Hospital for Sick Children, Toronto, Ontario, Canada. A Qubit Fluorometer High-Sensitivity Assay (Thermo Fisher Scientific) was used to measure DNA yield and a Nanodrop spectrophotometer (Thermo Fisher Scientific) was used to confirm DNA purity by measuring the ratio of absorbance at 260 nm and 280 nm. 400 ng of DNA sample was used for library preparation using the Illumina TruSeq PCR-free DNA Library Prep Kit. DNA was then sonicated into 350-bp fragments. A-tailed and indexed TruSeq Illumina adapters were ligated to end-repaired sheared DNA fragments before library amplification. Libraries were analyzed using Bioanalyzer DNA High-Sensitivity chips (Agilent Technologies, Santa Clara, CA, USA) and quantified using quantitative PCR. The libraries were loaded in equimolar quantities and pair-end sequenced on the Illumina HiSeqX platform to generate 150-bp reads. Integrative Genomics Viewer (IGV) version 2.8.2 was used for analysis with GRCm38/mm10 as the murine reference genome. Whole-genome sequencing for *Dmd* Δ52-54 and *Dmd* Δ52-55 mice was performed concurrently within the same sets of experiments and some results from these datasets for WT and *Dmd* Δ52-54 mice have been previously published in Wong et al. (2020).

### RNA extraction and sequencing

Mouse gastrocnemii were sectioned into 30-μm slices using a HM525 NX cryostat (Thermo Fisher Scientific), collected in 1.4 mm Zirconium Beads tubes (OPS Diagnostics) and homogenized using a MagNA Lyser (Roche Diagnostic). RNA extraction was performed using TRIzol reagent (Thermo Fisher Scientific) as per the manufacturer's protocol. Following RNA extraction, RNA was quantified using a Qubit RNA High-Sensitivity assay (Thermo Fisher Scientific). Sequencing was performed by The Centre for Applied Genomics using the Illumina HiSeq 2500 system, which produces 150-bp paired-end reads. Raw transcript reads were aligned to the GRCm38/mm10 mouse reference genome using STAR aligner, v.2.6.0c (https://github.com/alexdobin/STAR). HTSeq v.0.6.1p2 (https://htseq.readthedocs.io/en/master/history.html) was used to determine the absolute number of read counts for each gene. Normalization and differential expression analysis were completed using the R package DESeq2 v.1.26.0s (https://bioconductor.org/packages/release/bioc/html/DESeq2.html). An initial minimal filtering of ten reads per gene for all samples was applied to the datasets. Differentially expressed genes were defined as having a false discovery rate of <0.05 and fold change of >1.5 (for upregulated genes) or <0.6 (for downregulated genes). Gene Ontology analysis of the differentially expressed genes was performed using ShinyGo (http://bioinformatics.sdstate.edu/go74/) and gene set enrichment analysis was performed using the UC San Diego GSEA software (https://www.gsea-msigdb.org/gsea/index.jsp). Heat maps and volcano plots were made using GraphPad Prism v9. The RNA extraction and sequencing analyses for WT, *Dmd* Δ52-54 and *Dmd* Δ52-55 mice were performed concurrently within the same sets of experiments.

### Functional tests

Forelimb and hindlimb grip strength tests were conducted by The Centre for Phenogenomics based on the TREAT-NMD SOP DMD_M.2.2.001 protocol (https://www.treat-nmd.org/wp-content/uploads/2023/07/MDX-DMD_M.2.2.001.pdf). Mice were placed over the grid of the grip strength meter (Bioseb). Forepaws and hindpaws were allowed to attach to the grid before pulling the mouse back by the tail, and the maximal grip strength value of the mouse was measured. The grip strength test was done in triplicates, where the average grip strength value was normalized by mouse body weight.

For echocardiography, male mice were scanned using a Vevo2100 ultrasound machine (VisualSonics, Toronto, Canada) with a 30 MHz transducer as described previously (Zhou et al., 2005). All mice were scanned under 1.5% isoflurane anesthesia for 20-30 min with careful monitoring of the body temperature to maintain it at 37-38°C (TREAT-NMD DMD_M.2.2.003, https://www.treat-nmd.org/wp-content/uploads/2023/07/MDX-DMD_M.2.2.003.pdf). All measurements were conducted using the cardiac package of the Vevo 2100 v1.6.0 software.

For the open-field test, mice were placed in the frontal center of a transparent Plexiglas open field (41.25 cm×41.25 cm×31.25 cm) illuminated by 200 lux. The VersaMax Animal Activity Monitoring System (Omnitech Electronics; https://omnitech-usa.com/product/versamax-legacy-open-field/) recorded activity in the center and periphery of the open-field arena for 20 min per animal.

*In vivo* tibialis anterior contraction tests were performed as previously described (Kemaladewi et al., 2019). Briefly, contractile activity was measured using the 1300A: 3-in-1 Whole Animal System (Aurora Scientific) and analyzed using the Dynamic Muscle Analysis 5.5 and 5.3 high-throughput software (Aurora Scientific). The mice were anaesthetized using ketamine-xylazine solution at 100 mg/kg and 10 μl mg/kg to body weight, respectively, through intraperitoneal injection. Percutaneous electrodes were placed in the tibialis anterior and contractile output was measured.

For treadmilling exercises, 6-week-old C57BL/6J, *Dmd* Δ52-54 and *Dmd* Δ52-55 mice were placed on a treadmill (Columbus Instruments), which was set up at a downhill angle of 15° as previously described (Anderson et al., 2006). The mice were run for 10 min at 12 m/min for three consecutive days. The mice were subjected to a volitional fatigue protocol, where the objective was to fatigue but not exhaust them. Briefly, when the mice stopped running, they were prompted by the investigator to continue running until they no longer responded to the prompts. Although *Dmd* Δ52-55 and WT mice were able to finish the full protocol, *Dmd* Δ52-54 mice tended to finish the protocol earlier, on average. All exercised and sedentary mice were injected with EBD (Thermo Fisher Scientific) after the second day of exercise and *in vivo* contractile tests were performed after treadmill exercise on all mice after the third day of exercise. Muscle tissues were dissected 24 h post EBD injection.

The WT, *Dmd* Δ52-54 and *Dmd* Δ52-55 mouse functional data shown in Figs 3–5 were obtained concurrently within the same sets of experiments, and some results from these datasets for WT and *Dmd* Δ52-54 mice have been previously published in Wong et al. (2020), as indicated in the figure legends.

### Preparation and injection of EBD and assessment

EBD stock was prepared at 1% (w/v) in 1× phosphate-buffered saline (PBS) and filtered through Millex-GP 0.22 μm filters (Millipore, Bedford, MA, USA) (Hamer et al., 2002). Muscles dissected from mice injected with EBD were sectioned at 8 μm using a HM525 NX cryostat (Thermo Fisher Scientific) and mounted with ProLong Gold Antifade Mountant (Thermo Fisher Scientific). EBD-saturated myofibers fluoresce at 590 nm, and were scanned with the 3DHISTECH Panoramic Slide Scanner at the Imaging Facility at the Hospital for Sick Children and images were acquired with CaseViewer (3DHISTECH). Quantification was performed using ImageJ 1.52a software and the percentages of damaged fibers were calculated as the ratio of the EBD-positive area to the total tissue area.

### Tissue processing

Shortly following cervical dislocation, mouse hearts were arrested in diastole through direct KCl (1 M KCl in PBS) injection. All muscles dissected were frozen in cooled isopentane in liquid nitrogen as previously described (Nelson et al., 2016). Tissue processing for WT, *Dmd* Δ52-54 and *Dmd* Δ52-55 were performed concurrently within the same sets of experiments and some results from these datasets for WT and *Dmd* Δ52-54 mice have been previously published in Wong et al. (2020).

### Histological staining

All muscles were sectioned at 8 μm for histological staining. H&E staining was conducted using a standard protocol (Fischer et al., 2008). H&E slides were then scanned using the 3DHISTECH Pannoramic Slide Scanner by the Imaging Facility at the Hospital for Sick Children. CaseViewer (3DHISTECH) was used for image acquisition. Centralized nuclei were quantified using ImageJ 1.52a software from a total of 300 myofibers.

The WT, *Dmd* Δ52-54 and *Dmd* Δ52-55 mouse histological data shown in Fig. 2 were obtained concurrently within the same sets of experiments, and some results from these datasets for WT and *Dmd* Δ52-54 mice have been previously published in Wong et al. (2020), as indicated in the figure legends.

### Masson's trichome staining

Masson's trichrome staining was performed at the pathology laboratory at The Centre for Phenogenomics (TREAT-NMD SOP MDC1A_M.1.2.003, https://www.treat-nmd.org/wp-content/uploads/2023/07/cmd-MDC1A_M.1.2.003-67.pdf). Trichrome-stained slides were scanned using a Hamamatsu NanoZoomer and analyzed using the NDP.view2 viewing software (Hamamatsu). Three frames containing at least 300 fibers each were used for fibrosis quantification using ImageJ 1.51 software.

The WT, *Dmd* Δ52-54 and *Dmd* Δ52-55 mouse Masson's Trichrome data shown in Fig. 2 were obtained concurrently within the same sets of experiments, and some results from these datasets for WT and *Dmd* Δ52-54 mice have been previously published in Wong et al. (2020), as indicated in the figure legends.

### Immunofluorescence staining and analysis

8-μm sections of muscle tissues were used for immunofluorescence staining. Sections were fixed in ice-cold methanol and blocked with blocking buffer (3% normal goat serum and 0.2% bovine serum albumin in PBS). Primary antibodies were incubated overnight at 4°C. We used the following primary antibodies: rabbit polyclonal anti-dystrophin (ab15277; Abcam; 1:200), rabbit polyclonal anti-syntrophin-α1 (ab11187; Abcam; 1:600), rabbit polyclonal anti-β-sarcoglycan (ab222241; Abcam; 1:100) and rat monoclonal anti-laminin-2 (α2 chain; 4H8-2; Sigma-Aldrich; 1:500). The secondary antibodies used were Alexa Fluor 594-conjugated goat polyclonal anti-rabbit (A11012; Thermo Fisher Scientific; 1:250) and Alexa Fluor 488-conjugated goat polyclonal anti-rat (A11006; Thermo Fisher Scientific; 1:250) antibodies. Tissues were incubated at room temperature with secondary antibodies for 2 h. For sections stained with anti-laminin-α2, slides were scanned as described previously (Kemaladewi et al., 2017). Feret's diameter was quantified (TREAT-NMD SOP DMD-M1.2.001, https://www.treat-nmd.org/wp-content/uploads/2023/07/MDX-DMD_M.1.2.001.pdf) using Open-CSAM in ImageJ 1.51 software (Desgeorges et al., 2019).

The WT, *Dmd* Δ52-54 and *Dmd* Δ52-55 mouse immunofluorescence data shown in Fig. 1 were obtained concurrently within the same sets of experiments, and some results from these datasets for WT and *Dmd* Δ52-54 mice have been previously published in Wong et al. (2020), as indicated in the figure legends.

### Western blotting

We extracted proteins from homogenized mouse tissue by using a 1:1 solution of RIPA homogenizing buffer (50 mM Tris-HCl pH 7.4, 150 mM NaCl and 1 mM EDTA) and RIPA double-detergent buffer (2% deoxycholate, 2% NP-40 and 2% Triton X-100 in RIPA homogenizing buffer) supplemented with protease inhibitor cocktail (Roche), as described previously (Kemaladewi et al., 2019). The concentration of each protein was quantified using a Pierce BCA protein assay kit (Thermo Fisher Scientific). 15 μg of protein was prepared and western blotting was conducted using the NuPAGE electrophoresis system (Thermo Fisher Scientific). We used the following primary antibodies: mouse monoclonal anti-dystrophin (MANDYS8; Sigma-Aldrich; 1:5000), mouse monoclonal anti-vinculin (V284; Millipore; 1:2500) and mouse monoclonal anti-β-actin (sc-47778; Santa Cruz Biotechnology; 1:10,000).

The WT, *Dmd* Δ52-54 and *Dmd* Δ52-55 mouse western blot data shown in Fig. 1 were obtained concurrently within the same sets of experiments, and some results from these datasets for WT and *Dmd* Δ52-54 mice have been previously published in Wong et al. (2020), as indicated in the figure legends.

### Statistical analysis

GraphPad Prism version 7 was used to perform two-tailed unpaired Student's *t*-tests for all statistical analyses.

## Supplementary Material

10.1242/dmm.050595_sup1 Supplementary information
