# Supplementary material for: Generation and characterization of a mouse model of Becker muscular dystrophy with a deletion of Dmd exons 52 to 55
Source: Dis Model Mech. 2025 Sep 29;18(10):dmm050595. doi: 10.1242/dmm.050595 (PMC12519546; doi:10.1242/dmm.050595)
Supplement: Supplementary information [file dmm-18-050595-s1.pdf]

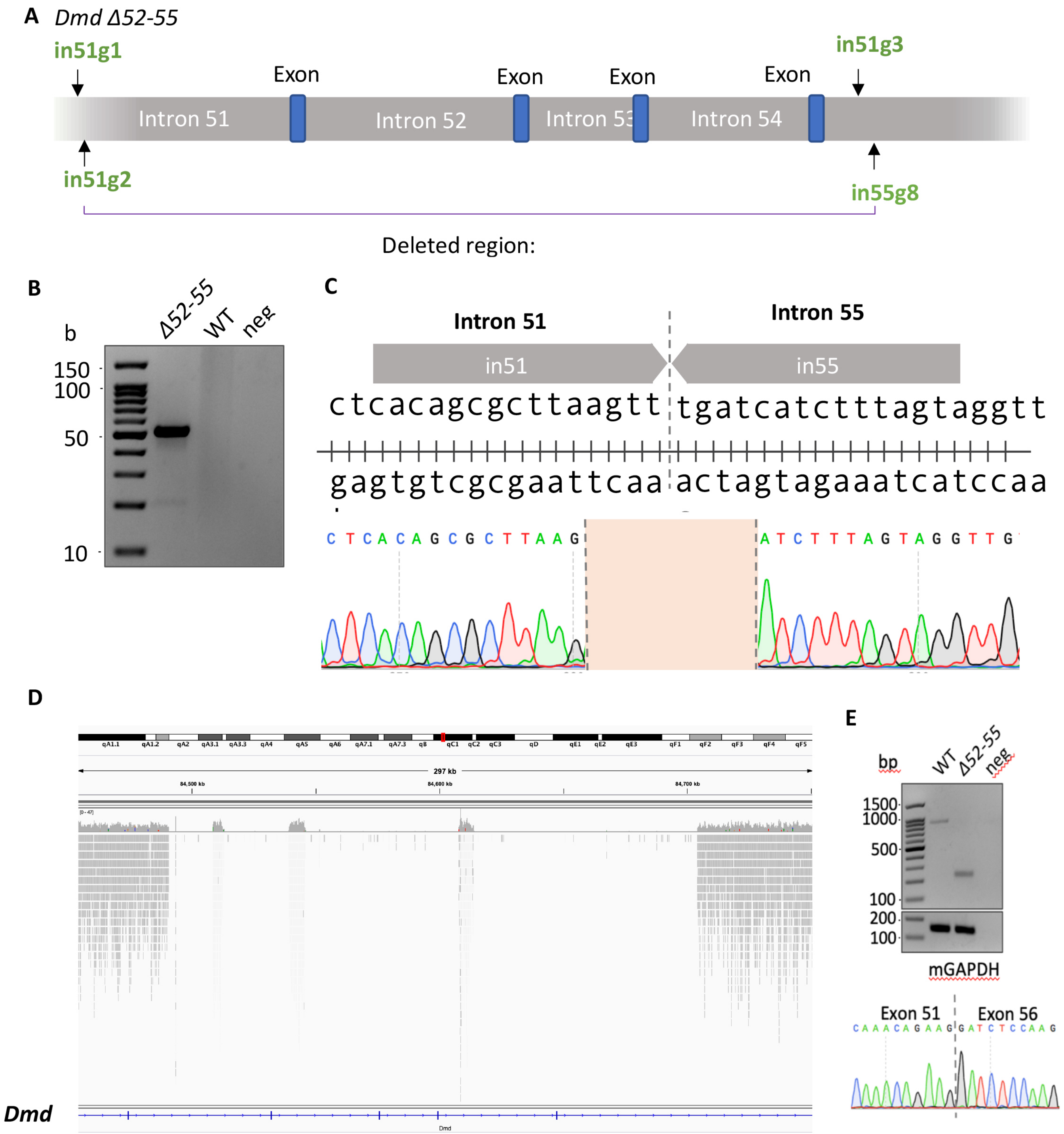

Disease Models & Mechanisms • Supplementary information

Fig. S1. Model generation. (A) Schematic showing sgRNAs that are compatible with a *Sp*Cas9 system and target introns 51 and 55, leading to a deletion of 213kb. (B, C) Genotyping detects the expected 213kb deletion junction. Sanger sequencing further reveals a 5 base pair deletion around the cut site. (D) Whole Genome Sequencing analysis confirmed the presence of the expected 213kb deletion spanning exons 52 to 55. (E) RT-PCR detection and Sanger sequencing of deletion junctions in *Dmd* transcripts in heart tissues of wildtype (WT), and *Dmd* del52-55 mice. No template was used in the sample called "neg".

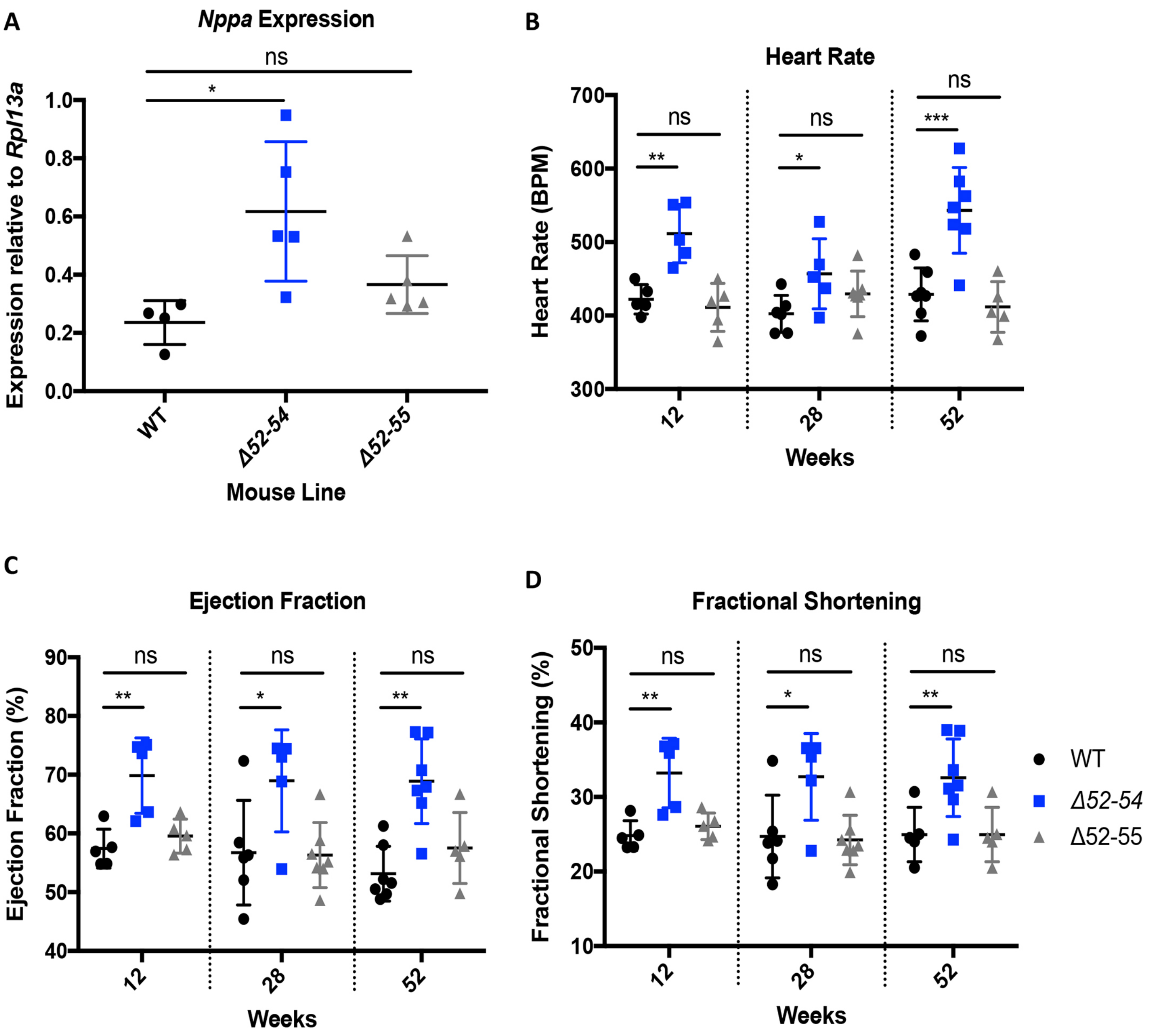

Fig. S2. *Dmd* del52-55 mice show little to no cardiac stress, similar to WT. (A) mRNA expression of *Nppa* in whole hearts at 12 weeks of age in WT and *Dmd* del52-55 mice. (B) Echocardiography was used to analyze the heart rate, (C) ejection fraction and (D) fractional shortening. All data is represented as ± S.D. Statistical analyses were performed with Student's t-test. P* < 0.05, P** < 0.01. The WT and *Dmd* del52-54 mouse data shown in panels A-D have been previously reported in Figs. 6 and S1 of Wong et al. (2020).

Disease Models & Mechanisms • Supplementary information

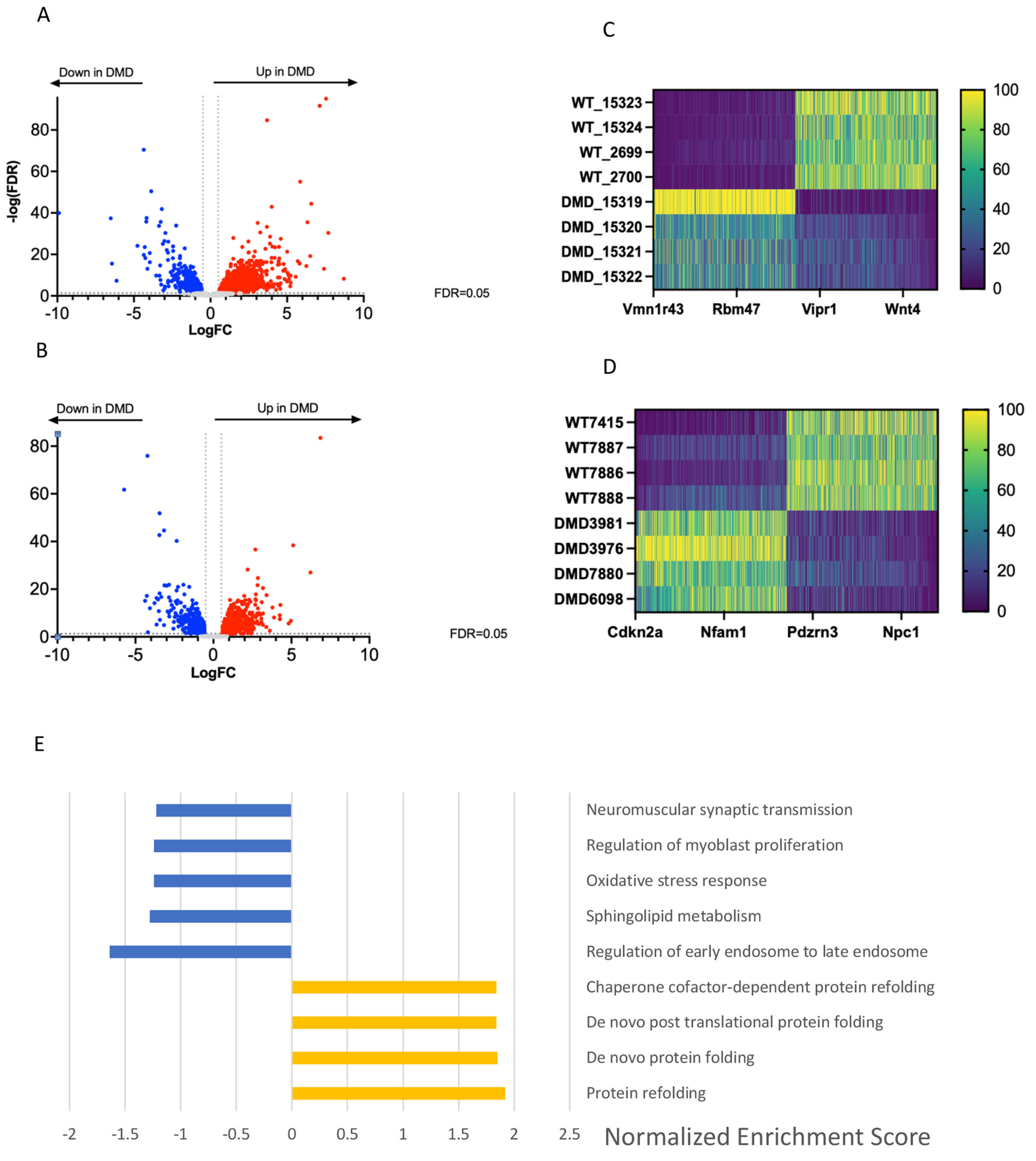
A
Down in DMD
Up in DMD
-log(FDR)
LogFC
FDR=0.05
B
Down in DMD
Up in DMD
LogFC
FDR=0.05
C
WT_15323
WT_15324
WT_2699
WT_2700
DMD_15319
DMD_15320
DMD_15321
DMD_15322
Vmn1r43
Rbm47
Vipr1
Wnt4
D
WT7415
WT7887
WT7886
WT7888
DMD3981
DMD3976
DMD7880
DMD6098
Cdkn2a
Nfam1
Pdzrn3
Npc1
E
Neuromuscular synaptic transmission
Regulation of myoblast proliferation
Oxidative stress response
Sphingolipid metabolism
Regulation of early endosome to late endosome
Chaperone cofactor-dependent protein refolding
De novo post translational protein folding
De novo protein folding
Protein refolding
Normalized Enrichment Score

Fig. S3. DMD mice harbor several differentially expressed genes compared to WT mice, pre- and post- exercise. (A) Volcano plot showing genes that are upregulated (in red) and downregulated (in blue) in *Dmd* del52-54 mice (n=4) compared to WT mice (n=4) after exercise. (B) Volcano plot showing genes that are upregulated (in red) and downregulated (in blue) in *Dmd* del52-54 mice (n=4) compared to WT mice (n=4) before exercise. (C) Heat map showing genes that are up- and down-regulated in *Dmd* del52-54 mice (n=4) compared to WT mice (n=4) after exercise. (D) Heat map showing genes that are up- and down-regulated in *Dmd* del52-54 mice (n=4) compared to WT mice (n=4) after exercise. (E) Gene Set Enrichment Analysis for 52 weeks old-exercised DMD (*Dmd* del52-54) and WT mice. Pathways in blue are downregulated while pathways in orange are upregulated.

Table S1. sgRNAs designed for the generation of *Dmd* Δ*52-54* and *Dmd* Δ*52-55* mouse lines

Distance Apart	Guide	Sequence (5’ > 3’)	PAM
316	In51g1	ACAGCGCTTAAGTTATCTGC	AGG
	In51g2	ATTGGTGGAGGGATTATATG	AGG
34	In54g3	GAAGGAATATAGGGTGATAG	AGG
	In54g2	GACTCATTGGAAGGAGTGAT	GGG
89	In55g8	CCTACTAAAGATGATCAGCT	AGG
	In55g3	TAAACTTACAACCTAGTCAC	AGG

Table S2. Oligonucleotides utilized for molecular analyses

Genotyping *Dmd Δ52- 55*	M_in51-2-F	AGTACCATTGTCCCATATGTACATG
	M_in55-1-R	GACTTTTGGGATAGCATCCTAAATG
qRT-PCR-*Nppa* expression	*Nppa* Fwd	GGGTAGGATTGACAGGATTGG
	Nppa Rvrse	CTCCTTGGCTGTTATCTTCGG
	Rp113a Fwd	GAAGGCTAAGATCCACTACCG
	Rp113a Rvrse	TTTATTGTGCTCAGACCAGGAG

Disease Models & Mechanisms • Supplementary information
